# New gorilla adenovirus vaccine vectors induce potent immune responses and protection in a mouse malaria model

**DOI:** 10.1186/s12936-017-1911-z

**Published:** 2017-07-03

**Authors:** Keith Limbach, Maureen Stefaniak, Ping Chen, Noelle B. Patterson, Grant Liao, Shaojie Weng, Svetlana Krepkiy, Greg Ekberg, Holly Torano, Damodar Ettyreddy, Kalpana Gowda, Sharvari Sonawane, Arnel Belmonte, Esteban Abot, Martha Sedegah, Michael R. Hollingdale, Ann Moormann, John Vulule, Eileen Villasante, Thomas L. Richie, Douglas E. Brough, Joseph T. Bruder

**Affiliations:** 1 Malaria Department, Naval Medical Research Center, 503 Robert Grant Avenue, Silver Spring, MD USA; 2 Henry M. Jackson Foundation for the Advancement of Military Medicine, Inc., 6720A Rockledge Drive, Suite 100, Bethesda, MD USA; 3 GenVec Incorporated, 910 Clopper Road, Suite 220N, Gaithersburg, MD USA; 4 University of Massachusetts Medical School, Worcester, MA USA; 5 Center for Global Health Research, Kenya Medical Research Institute, Kisumu, Kenya

**Keywords:** Genetic, Molecular, Vaccine, Malaria, Adenovector, Adenovirus, Gorilla, Non-human primate, Single-administration, Efficacy

## Abstract

**Background:**

A DNA-human Ad5 (HuAd5) prime-boost malaria vaccine has been shown to protect volunteers against a controlled human malaria infection. The potency of this vaccine, however, appeared to be affected by the presence of pre-existing immunity against the HuAd5 vector. Since HuAd5 seroprevalence is very high in malaria-endemic areas of the world, HuAd5 may not be the most appropriate malaria vaccine vector. This report describes the evaluation of the seroprevalence, immunogenicity and efficacy of three newly identified gorilla adenoviruses, GC44, GC45 and GC46, as potential malaria vaccine vectors.

**Results:**

The seroprevalence of GC44, GC45 and GC46 is very low, and the three vectors are not efficiently neutralized by human sera from Kenya and Ghana, two countries where malaria is endemic. In mice, a single administration of GC44, GC45 and GC46 vectors expressing a murine malaria gene, *Plasmodium yoelii* circumsporozoite protein (*Py*CSP), induced robust *Py*CSP-specific T cell and antibody responses that were at least as high as a comparable HuAd5-*Py*CSP vector. Efficacy studies in a murine malaria model indicated that a prime-boost regimen with DNA-*Py*CSP and GC-*Py*CSP vectors can protect mice against a malaria challenge. Moreover, these studies indicated that a DNA-GC46-*Py*CSP vaccine regimen was significantly more efficacious than a DNA-HuAd5-*Py*CSP regimen.

**Conclusion:**

These data suggest that these gorilla-based adenovectors have key performance characteristics for an effective malaria vaccine. The superior performance of GC46 over HuAd5 highlights its potential for clinical development.

**Electronic supplementary material:**

The online version of this article (doi:10.1186/s12936-017-1911-z) contains supplementary material, which is available to authorized users.

## Background

Malaria, HIV1 and TB kill more than four million people every year [1]. Highly effective vaccines against these diseases have proven difficult to develop and are not yet available. Although most licensed vaccines work by inducing efficacious antibody responses [2], data from numerous preclinical and clinical studies suggest that highly effective vaccines against these three pathogens may need to elicit strong T cell responses [3–9]. Adenovirus vectors are highly effective vaccine platforms for inducing potent CD8^+^ T cell responses [10]. Therefore, adenovirus vectors represent attractive vaccine platforms for these pathogens.

CD8^+^ T lymphocytes are important mediators of protective immunity against the malaria liver stage [11–20]. A gene-based vaccine approach was developed based on findings that heterologous prime-boost immunization induces CD8^+^ T cells and protection against malaria in mice [21–24], non-human primates [25] and humans [5, 6, 26–31]. Particularly encouraging data were obtained in a clinical study using a DNA prime-human adenovirus serotype 5 (HuAd5) boost regimen expressing two malaria antigens, *Plasmodium falciparum* circumsporozoite protein (*Pf*CSP) and *P. falciparum* apical membrane antigen 1 (*Pf*AMA1). In this study, 4 of 15 volunteers were sterilely protected against controlled human malaria infection (CHMI) [5]. CD8^+^ T cell responses specific for *Pf*AMA1 were associated with protection, and two of the four protected volunteers had the highest frequency of CD8^+^ T cell responses specific for *Pf*CSP [5]. DNA priming was essential as the HuAd5 vectors alone failed to elicit protection [5, 32].

Although these results are encouraging, they do not meet the preferred product characteristics target of 75% efficacy set forth by the World Health Organization (WHO) [33]. One approach to improve vaccine efficacy is to identify more potent adenovirus vaccine vectors.

One factor that can affect the potency of an adenovirus-based vaccine is pre-existing immunity against the adenovirus vector. HuAd5 is a relatively common human pathogen, with 35–50% seroprevalence in the USA and Europe and 70–95% seroprevalence in malaria-endemic regions of sub-Saharan Africa [34–39]. Pre-existing immunity against HuAd5 can affect the immunogenicity and efficacy of HuAd5-based vaccines, reducing the percentage of responders and the frequency of T cell responses [40–42]. In the DNA-HuAd5 malaria trial, 50% of the volunteers with low (<12) HuAd5 pre-existing neutralizing antibodies (NAb) titres were protected against CHMI, whereas none of the volunteers with high (>500) pre-existing NAb titres were protected [5]. Since the vast majority of malaria-related deaths occur in sub-Saharan Africa, where the HuAd5 seroprevalence is very high, pre-existing HuAd5 immunity could have a dramatic impact on the efficacy of a HuAd5-based malaria vaccine.

A second factor that can affect the potency of an adenovirus-based vaccine is the inherent capacity of specific adenovirus serotypes to induce immune responses to the encoded transgene. Most studies indicate that HuAd5 and chimpanzee adenovirus type 3 (ChAd3) induce very robust transgene-specific immunity and that other leading adenovirus vectors are less immunogenic and less protective in animal models [39, 43–46]. These studies highlight the need for identifying new highly immunogenic adenovirus serotypes with low seroprevalence in human populations for vaccine development.

Three gorilla adenoviruses, GC44, GC45, and GC46, are being developed as vaccine vectors. Although the gorilla adenoviruses are closely related to human species C adenoviruses [47], they are not efficiently neutralized by human sera. Less than 6% of people living in the United States are seropositive for GC44, GC45 or GC46; and the few individuals who are seropositive have very low NAb titres against these viruses [38]. In this report, the immunogenicity and efficacy of GC44, GC45 and GC46 vectors expressing the *P. yoelii* circumsporozoite protein (*Py*CSP) are described. One vector, GC46, was more efficacious than HuAd5 in this murine malaria model, suggesting that GC46 is a strong candidate for further malaria vaccine development.

## Methods

### Adenovirus seroprevalence and neutralizing antibody assay

NAb assays were performed as previously described [38] . In brief, serum samples from adults residing in the USA (n = 250), Kenya (n = 90) and Ghana (n = 100) were diluted 1:16, incubated with HuAd5, GC44, GC45 and GC46 vectors expressing the firefly luciferase gene for 1 h at room temperature, and then used to infect 5 × 10^4^ A549 cells in triplicate at a multiplicity of infection (MOI) of 2000 virus particle units (pu)/cell. Twenty-four hours post-infection, the cells were lysed with Cell Culture Lysis Buffer (Promega, Madison, WI) and luciferase activity was measured using the Luciferase Assay Reagent System (Promega). Samples that resulted in >90% reduction in luciferase activity compared to the virus-only control were defined as positive for NAb. The positive samples were then diluted from 1:32 to 1:1024 in twofold increments and tested for their capacity to neutralize the same set of vectors. The endpoint titre was defined as the maximum dilution at which the serum sample displayed a 90% reduction in luciferase activity compared to the virus-only control.

### DNA and adenovirus vaccine vectors

#### Plasmid DNA vectors

The DNA-*Py*CSP vector (VR2516) was generated by cloning the native, full-length *Py*CSP gene into VR1020 (Vical, San Diego, CA) [48]. This cloning reaction positions the *Py*CSP gene downstream from a human cytomegalovirus (HCMV) Immediate-Early (IE) promoter and in-frame with a human tissue plasminogen activator (TPA) signal sequence [48]. The DNA Null vector (VR1020) contains a TPA signal sequence, but does not contain a transgene. VR2516 and VR1020 were manufactured to preclinical grade specifications (Premium Research *Ready* plasmid DNA) by Puresyn, Inc. (Malvern, PA).

#### Adenovirus vectors

The E1-, partial E3-, E4-deleted, replication-incompetent HuAd5-*Py*CSP vector and the E1-deleted, replication-incompetent GC44-*Py*CSP, GC45-*Py*CSP and GC46-*Py*CSP vectors were generated in ORF6 cells [49] using a plasmid-based construction system that positions the *Py*CSP gene in the E1 region downstream from a HCMV IE promoter [24]. The HuAd5-*Py*CSP, GC44-*Py*CSP, GC45-*Py*CSP and GC46-*Py*CSP vectors express a codon-optimized *Py*CSP gene in which the *Py*CSP glycosylphosphatidylinositol (GPI) anchor has been deleted. The HuAd5 Null and GC46 Null vectors do not express a transgene.

### Mice and parasites

Six week old female BALB/c mice were purchased from either Harlan Laboratories (Frederick, MD) or the National Cancer Institute (Frederick, MD). *Plasmodium yoelii* (17XNL non-lethal strain) parasites were maintained by alternating passage in *Anopheles stephensi* mosquitoes and female CD1 outbred mice.

Female BALB/c mice were injected intramuscularly in the tibialis anterior muscle with 100 µl of vaccine (50 µl in each leg). The DNA vectors were prepared and diluted for immunization in 1× phosphate buffered saline (PBS). The adenovirus vectors were prepared and diluted for immunization in final formulation buffer [24]. In the single-dose immunogenicity study, 6 mice/group were immunized with 1 × 10^7^, 1 × 10^8^ or 1 × 10^9^ pu of HuAd5-*Py*CSP, GC44-*Py*CSP, GC45-*Py*CSP, GC46-*Py*CSP or HuAd5 Null. Twenty-one days post-immunization, the mice were killed and splenocytes and sera harvested for evaluation of *Py*CSP-specific T cell and antibody responses.

In protection study 1, 20 mice/group were primed on day 0 with 100 µg of DNA-*Py*CSP (VR2516) and boosted on day 43 with 1 × 10^9^ pu of HuAd5-*Py*CSP, GC44-*Py*CSP, GC45-*Py*CSP or GC46-*Py*CSP. On days 38 and 52, the mice were bled and sera prepared for evaluation of *Py*CSP-specific antibody responses. On day 55, 6 mice/group were killed and splenocytes harvested for evaluation of *Py*CSP-specific T cell responses. On day 57, 14 mice/group were challenged intravenously in the tail vein with 100 *P. yoelii* sporozoites isolated from the salivary glands of infected mosquitoes and diluted for challenge in M199 medium containing 5% normal mouse serum. On days 62–71, parasitaemia was evaluated by examining Giemsa-stained thin blood smears. In protection studies 2 and 3, mice were boosted with HuAd5-*Py*CSP or GC46-*Py*CSP, but not with GC44-*Py*CSP or GC45-*Py*CSP. Otherwise, the experimental design of the three protection studies was similar. Each protection study contained a group of 20 naïve, non-immunized mice and a group of 20 negative control mice that were primed with VR1020 and boosted with GC46 Null. Mice were considered positive if blood stage parasites were observed in any blood smear. To gauge the severity of the challenge, an ID_50_ was determined by challenging four groups of naïve BALB/c mice (6 mice/group) with four suboptimal doses of *P. yoelii* sporozoites (33, 11, 3.7 or 1.2 sporozoites). (An ID_50_, or infectious dose 50, represents the dose of sporozoites required to infect 50% of challenged mice.) From these infectivity control mice, an ID_50_ for protection studies 1, 2 and 3 was calculated to be 2.45 sporozoites, 3.4 sporozoites and 3.4 sporozoites, respectively.

### Splenocytes

Single cell splenocyte suspensions were prepared by gently crushing the spleen between a 70 µm cell strainer placed over a 50 ml conical tube and the flat end of a sterile 3 ml syringe plunger while rinsing the cells with cold wash buffer (1× Hank’s Balanced Salt Solution without Ca^2+^ and Mg^2+^, with 0.5% FBS and 10 mM HEPES). The cell suspension was washed twice with wash buffer, then the cell pellet was resuspended in 5 ml of Red Blood Cell Lysis Buffer (Sigma-Aldrich, St. Louis, MO). Following a 3 min lysis, wash buffer was added to a final volume of 50 ml. The cell suspension was then pelleted, resuspended in 20 ml of R10 media (RPMI-1640 media with 10% FBS, 1× GlutaMax™-1 Supplement and 1× Penicillin–Streptomycin), passed through a second 70 µm cell strainer into a clean 50 ml conical tube, counted with a Guava PCA (EMD Millipore, Billerica, MA), pelleted and resuspended in R10 media at a final concentration of 1 × 10^7^ cells/ml.

### Stimulator cells

Peptide-pulsed stimulator cells were prepared by pulsing A20.2J (Clone HB-98, ATCC, Manassas, VA) suspension cells (1 × 10^7^ cells/ml) with peptides (20 µg/ml for peptides <10 amino acids and 100 µg/ml for peptides >10 amino acids) for a minimum of 1 h with gentle mixing every 20 min. The peptide-pulsed A20.2J cells were irradiated in a Cobalt-60 irradiator (16,666 rad), washed with R10 media and resuspended in R10 media at a final concentration of 1.35 × 10^6^ cells/ml for the ELISpot assays, or 1.5 × 10^6^ cells/ml for the ICS assays. Additional peptide was added to the cell suspension at a final concentration of 20 µg/ml.

### IFN-γ ELISpot

IFN-γ ELISpot responses were assessed with fresh splenocytes in group pools (6 mice/group) in quadruplicate wells. Group pools were prepared by combining splenocytes from the individual mice in equal ratios. Splenocytes were stimulated with A20.2J cells pulsed with peptides encoding the *Py*CSP immunodominant CD8^+^ T cell epitope (amino acids 280–288, SYVPSAEQI) [50] or the *Py*CSP CD4^+^ immunodominant/nested CD8^+^ subdominant T cell epitopes (amino acids 57–70, KIYNRNIVNRLLGD) [50] (AnaSpec, Fremont, CA). Media alone, splenocytes stimulated with irradiated non-pulsed A20.2J cells and PMA/Ionomycin-stimulated splenocytes served as assay controls. In brief, multiscreen MSHAS4510 plates (EMD Millipore) were coated with 100 µl/well of a rat anti-mouse IFN-γ antibody (Clone R4-6A2, BD Biosciences, San Jose, CA) at a final concentration of 10 µg/ml in 1× PBS, pH 7.2. The plates were washed 3 times with RPMI-1640 media and blocked with R10 media at 37 °C in 5% CO_2_ for a minimum of 3 h. The blocking media were then removed and the splenocyte group pools were plated at 400,000, 200,000 and 100,000 cells/well with 135,000 peptide-pulsed A20.2J stimulator cells/well. The plates were incubated at 37 °C in 5% CO_2_ for 40 h and washed 9 times with PBS-T buffer (1× PBS, pH = 7.2 with 0.01% Tween-20). Biotinylated rat anti-mouse IFN-γ antibody (clone XMG1.2, BD Biosciences) at 1 µg/ml in 1× PBS, pH = 7.2 was then added and the plates were incubated for 3 h at room temperature. After 3 washes with PBS-T, the plates were incubated for 1 h at room temperature with a 1:800 dilution of peroxidase-labeled streptavidin (KPL, Gaithersburg, MD) in 1× PBS, pH = 7.2. The plates were then washed 3 times with PBS-T and 3 times with 1× PBS, pH = 7.2. The spots were developed with DAB Reagent Set (KPL) according to the manufacturer’s instructions. The spots were counted using an AID ELISpot Reader (Autoimmun Diagnostika, Strassberg, GER). Data analysis was done with Microsoft Excel 2008 for Mac (Microsoft, Redmond, WA) and GraphPad Prism v5.0c (GraphPad Software, La Jolla, CA).

### Flow cytometry: intracellular cytokine (ICS) staining and cell phenotyping

Freshly isolated splenocytes from individual mice (6 mice/group) were stimulated with A20.2J cells pulsed with peptides encoding the *Py*CSP immunodominant CD8^+^ T cell epitope (amino acids 280–288, SYVPSAEQI), the *Py*CSP CD4^+^ immunodominant/nested CD8^+^ subdominant T cell epitopes (amino acids 57–70, KIYNRNIVNRLLGD) or the influenza hemagglutinin CD8^+^ T cell epitope (amino acids 332–340, TGLRNTPSI). Media, splenocytes stimulated with irradiated non-pulsed A20.2J cells and PMA/Ionomycin-stimulated splenocytes served as assay controls. In brief, 1 × 10^6^ splenocytes from individual mice and 1.5 × 10^5^ peptide-pulsed A20.2J stimulator cells were incubated for 6–8 h at 37 °C in 5% CO_2_ in 96-well round bottom plates. BD Golgi Plug™ (BD Bioscience) was added 1 h into the incubation to block cytokine release. The plates were then wrapped in plastic wrap and stored at 4 °C overnight. The samples were stained for viability using the LIVE/DEAD^®^ Fixable Blue Dead Cell Stain Kit for UV excitation from Molecular Probes^®^ (Life Technologies, Grand Island, NY) and blocked for non-specific staining using Mouse BD Fc Block™ (BD Biosciences). For the single-dose immunogenicity study, the samples were surface-stained with the following antibodies (fluorochrome): CD4—RM4-5(eFlur-450) (eBioscience, San Diego, CA) and CD8a—53-6.7(PerCP-Cy5.5) (BD Biosciences). Following separate fixation and permeabilization steps, the samples were stained intracellularly with the following antibodies (fluorochrome): IFN-γ—XMG1.2(PE), TNF-α—MP6-XT22(APC), and IL-2—JES6-5H4 (Alexa488) (BD Biosciences). The data were acquired using a BD FACSCalibur E1610 equipped with a Cytek DxP Multi-Colour Upgrade (using 3 lasers to allow acquisition of 8 colours) and an Automated Micro-Sampler (96 well format) (BD Biosciences). The samples for the prime-boost experiments were surface-stained with the following antibodies (fluorochrome): CD19—1D3 (APC-H7), CD4—RM4-5(V500) (BD Biosciences), NKp46—29A1.4 (PerCP-e710) (eBioscience San Diego, CA), CD44—IM7(PE-Cy7), CD127—A7R34(BV421) and KLRG1—2F1/KLRG1(APC) (Biolegend, San Diego, CA). Following separate fixation and permeabilization steps, the samples were stained intracellularly with the following fluorochrome-labeled antibodies: CD8a—53-6.7(BV785), TNF-α—MP6-XT22(BV605) (Biolegend), CD3e-500A2 (Alexa 700), IFN-γ—XMG1.2(Alexa 488) and IL-2—JES6-5H4(PE) (BD Biosciences). The data were acquired using a BD LSR II equipped with 4 lasers and the automated 96-well high throughput system (BD Biosciences). Primary data analysis was done with FlowJo v9.5.2 (Tree Star, Ashland, OR). Subsequent data analyses were done with Microsoft Excel 2008 for Mac and GraphPad Prism v5.0c.

### ELISA

In the single-dose immunogenicity study, Immulon 4 HBX flat-bottom microtitre plates (Dynex Technologies, Chantilly, VA) were coated overnight with purified *Py*CSP protein produced in yeast at a final concentration of 0.1 µg/ml. In the prime-boost protection studies, Immulon 4 HBX plates were coated with a synthetic peptide encoding a portion of the immunodominant *Py*CSP repeat region (QGPGAP)_4_ (AnaSpec) at a final concentration of 0.5 µg/ml (0.05 µg/well) in coating buffer (15 mM sodium carbonate, 35 mM sodium bicarbonate in UltraPure Water). The plates were blocked for 2 h with casein blocker in Tris buffered saline (TBS) (Pierce Thermo Scientific, Rockford, IL), washed with 1× TBS with 0.1% Tween-20 and incubated for 2 h at room temperature with threefold serial dilutions of sera from individual mice in triplicate wells. The plates were then washed and incubated for 2 h with a 1:2000 dilution of ReserveAP phosphatase-labeled goat anti-mouse IgG (H + L), human serum absorbed (KPL) in Diluting Buffer (1× TBS with 0.1% BSA, 0.05% Tween-20). The plates were washed and developed with 0.1 mg/well of *p*-Nitrophenyl phosphate (Sigma-Aldrich) dissolved in Coating Buffer. The reaction was stopped by adding 25 µl/well of stop solution (5 N Sodium Hydroxide). Absorbance was measured at 405 nm using a SpectraMax 190 ELISA reader (Molecular Devices, Sunnyvale, CA) or a SpectraMax 340PC plate reader (Molecular Devices). Data analysis was done with Microsoft Excel 2008 for Mac and GraphPad Prism v5.0c.

### Indirect immunofluorescent antibody (IFA)

Sporozoite stage IFAs were performed as previously described [51] using a fluorescein isothiocyanate (FITC)-conjugated goat anti-mouse IgG antibody (KPL). Endpoint dilutions were determined for individual mice, except for the negative control and naïve samples, where group pools were used. Endpoint dilution values represent the average of two separate assays.

### Statistical analysis

Statistical analysis of the single-dose immunogenicity data was performed using one-way analysis of variance (ANOVA) followed by Bonferroni’s mean comparison test with Origin Pro 8.0 (OriginaLab, Northhampton, MA). Statistical analysis of the protection data was performed using a two-tailed Fisher’s Exact Test with GraphPad Prism v5.0c. Statistical analysis of the prime-boost ICS data was performed using a non-parametric, two-tailed, Mann–Whitney *U* test with GraphPad Prism v5.0c. Statistical analysis of the prime-boost ELISA data was performed using an unpaired, two-tailed *t* test with GraphPad Prism v5.0c. Statistical analysis of the IFA data was performed using a two-tailed *t* test with GraphPad Prism v5.0c. *P* values of less than 0.05 were considered significant.

## Results

### Seroprevalence of GC44, GC45 and GC46 is low in humans living in Kenya and Ghana

**Fig. 1:**
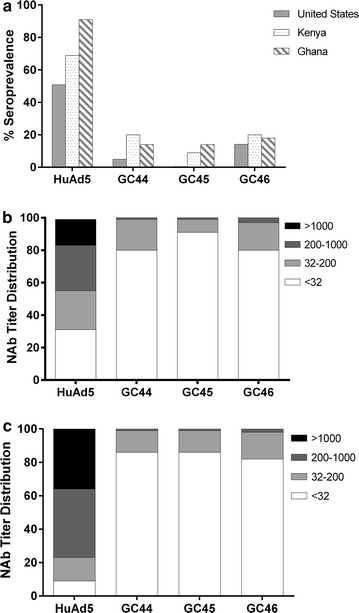
Seroprevalence of GC44, GC45 and GC46 in North American and African adults. **a** The seroprevalence (NAb titre >32) of HuAd5, GC44, GC45 and GC46 was determined in 250 adults living in the United States, 90 adults in Kenya and 100 adults in Ghana. In Kenya, the seroprevalence was 69, 20, 9 and 20% for HuAd5, GC44, GC45 and GC46, respectively. In Ghana, the seroprevalence was 91, 14, 14 and 18% for HuAd5, GC44, GC45 and GC46, respectively. The NAb titres of individuals from Kenya (**b**) and Ghana (**c**) were segregated into four categories; <32, 32–200, 200–1000 or >1000. Higher NAb titres are represented by progressively darker colours. 16% of the Kenyan samples and 36% of the Ghanaian samples had NAb titres against HuAd5 that were >1000, whereas none of the samples had comparable titres against GC44, GC45 or GC46. Additionally, 44% of the Kenyan samples and 77% of the Ghanaian samples had NAb titres against HuAd5 that were >200, whereas only 1, 1 and 3% of the Kenyan samples and 1, 1 and 2% of the Ghanaian samples had comparable titres against GC44, GC45 and GC46

Since the largest target population for a malaria vaccine resides in sub-Saharan Africa, the seroprevalence of GC44, GC45 and GC46 in sera samples from adults living in Kenya and Ghana were evaluated, and these outcomes were compared with the seroprevalence of HuAd5. Consistent with results from the USA, the seroprevalence of the gorilla adenoviruses was much lower than HuAd5 in Kenya and Ghana (Fig. 1a). Moreover, in the few seropositive individuals, the NAb titres against GC44, GC45 and GC46 were much lower than the NAb titres against HuAd5. Specifically, only 1–3% of individuals from both African countries had titres >200 and none had titres >1000 (Fig. 1b, c). Overall, these results indicate a low prevalence of neutralizing antibodies to GC44, GC45 and GC46 in malaria-endemic regions of sub-Saharan Africa, suggesting that they are suitable candidate vectors for malaria vaccine development.

### GC44, GC45 and GC46 vectors expressing *Py*CSP induce robust *Py*CSP-specific T cell and antibody responses in mice

**Fig. 2:**
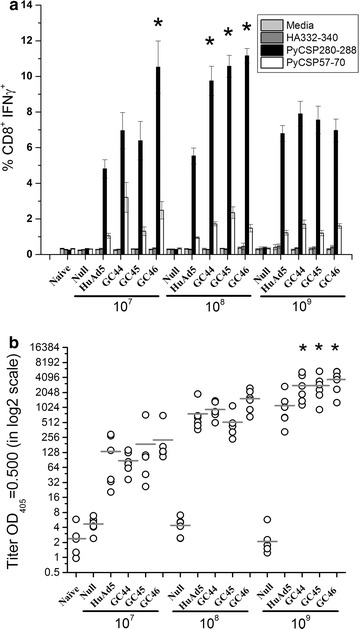
T cell and antibody responses induced by a single immunization of HuAd5-*Py*CSP, GC44-*Py*CSP, GC45-*Py*CSP or GC46-*Py*CSP. BALB/c mice (6 mice/group) were immunized with three different doses (1 × 10^7^, 1 × 10^8^ or 1 × 10^9^ pu) of HuAd5 Null, HuAd5-*Py*CSP, GC44-*Py*CSP, GC45-*Py*CSP or GC46-*Py*CSP. **a** CD8^+^ T cell IFN-γ responses were assessed 21 days after immunization by ICS. Splenocytes were stimulated with A20.2J cells loaded with peptides encoding the *Py*CSP immunodominant CD8^+^ T cell epitope (amino acids 280–288), the *Py*CSP immunodominant CD4^+^/nested CD8^+^ subdominant T cell epitopes (amino acids 57–70) or the influenza hemagglutinin CD8^+^ T cell epitope (amino acids 332–240). *Error bars* indicate standard errors of the means (n = 6). *p <0.05 compared with HuAd5 at the same dose. **b** *Py*CSP-specific antibody responses were assessed 21 days after immunization by ELISA. The capture antigen was purified *Py*CSP protein. The values for individual mice are shown (n = 6). The mean is indicated by the *black line*. *p <0.05 compared with HuAd5 at the same dose

To further evaluate the vaccine potential of the three gorilla adenoviruses, the immunogenicity of E1-deleted, replication-incompetent GC44, GC45 and GC46 vectors that express *Py*CSP was evaluated in BALB/c mice (Fig. 2). Mice were immunized with three different doses of each vector. Positive control mice were immunized with comparable doses of a HuAd5-*Py*CSP vector and negative control mice were immunized with comparable doses of a HuAd5 Null vector that does not express a transgene. Three weeks after immunization, *Py*CSP-specific T cell and antibody responses were evaluated by intracellular cytokine staining (ICS) and ELISA assays. Each GC-*Py*CSP vector induced strong *Py*CSP-specific CD8^+^ T cell and antibody responses (Fig. 2). At the two lower doses (1 × 10^7^ and 1 × 10^8^ pu), the three GC-*Py*CSP vectors each induced a higher percentage of IFN-γ-positive CD8^+^ T cells than HuAd5-*Py*CSP. At the highest dose (1 × 10^9^ pu), the GC-*Py*CSP and HuAd5-*Py*CSP vectors induced comparable responses (Fig. 2a). The GC-*Py*CSP vectors also induced higher antibody responses than HuAd5-*Py*CSP, especially at the 1 × 10^9^ pu dose, where the differences were statistically significant (p < 0.05) (Fig. 2b). Overall, these results indicate that GC44, GC45 and GC46 are highly immunogenic and induce responses equal to or higher than HuAd5.

### DNA-GC46 vaccine is more efficacious than DNA-HuAd5 vaccine in the *P. yoelii*/mouse malaria model

**Fig. 3:**
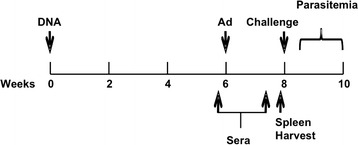
Design of DNA prime-Ad boost protection studies. On week 0, BALB/c mice (20 mice/group) were primed with 100 µg of DNA-*Py*CSP. On week 6, the mice were boosted with 1 × 10^9^ pu of HuAd5-*Py*CSP, GC44-*Py*CSP, GC45-*Py*CSP or GC46-*Py*CSP (protection study 1), or 1 × 10^9^ pu of HuAd5-*Py*CSP or GC46-*Py*CSP (protection studies 2 and 3). Five days before (study day 38) and 9 days after (study day 52) the adenovirus boost, the mice were bled and sera isolated for antibody studies. Twelve days after the boost, 6 mice/group were euthanized and splenocytes isolated for T cell studies. On week 8, 14 mice/group were challenged with 100 *P. yoelii* sporozoites. Blood stage parasitaemia was evaluated 5–14 days post-challenge

The efficacy of the GC-*Py*CSP vectors was evaluated in the *P. yoelii*/mouse malaria model using a DNA-adenovirus prime-boost regimen. Three separate protection studies were performed. The general design of these studies is illustrated in Fig. 3. In the first study, all three GC vectors were compared with HuAd5. Since the highest levels of efficacy were achieved with GC46, this vector was selected for subsequent experiments (Table 1).

**Table 1:** Efficacy results

Vaccine	Protection^a^ Protected mice/total mice (% protected)				Statistical analyses	
					p value	
	Study 1	Study 2	Study 3	Total	Total vs. Null	Total vs. DNA-HuAd5
DNA-HuAd5	5/14 (36%)	2/14 (14%)	3/14 (21%)	10/42 (24%)	0.0011^b^	–
DNA-GC44	6/14 (43%)	–	–	6/14 (43%)	0.0159^c^	1.000^c^
DNA-GC45	2/14 (14%)	–	–	2/14 (14%)	0.4815^c^	0.3845^c^
DNA-GC46	7/14 (50%)	5/14 (36%)	13/14 (93%)	25/42 (60%)	<0.0001^b^	0.0018^b^
Null	0/14 (0%)	0/14 (0%)	0/14 (0%)	0/42 (0%)	–	
Naïve	0/14 (0%)	0/14 (0%)	0/14 (0%)	0/42 (0%)		

^a^The number and percentage of mice protected in each study, as well as the total number and percentage of mice protected in all three studies is presented. Statistical analyses (two-tailed Fisher’s Exact Test) utilized data from all three studies (n = 42)^b^ or data from protection study 1 (n = 14)^c^

In study 1, the efficacy of the DNA-GC44 and DNA-GC46 vaccines was slightly higher than the DNA-HuAd5 vaccine, and much higher than the DNA-GC45 vaccine (Table 1). None of the 14 mice immunized with the DNA and GC46 Null vectors, and none of the 14 naïve mice were protected (Table 1). The protection induced by the DNA-HuAd5 vaccine (p = 0.0407), DNA-GC44 vaccine (p = 0.0159) and DNA-GC46 vaccine (p = 0.0058), but not the DNA-GC45 vaccine (p = 0.4815), was statistically significant compared to the negative controls.

To confirm the higher efficacy of the DNA-GC46 vaccine relative to the DNA-HuAd5 vaccine, two further studies were performed with the DNA-HuAd5 and DNA-GC46 vaccines. In study 2, the efficacy of the DNA-HuAd5 vaccine was lower than in study 1 and was not statistically significant compared to the negative controls (p = 0.4815). In contrast, the efficacy of the DNA-GC46 vaccine was higher than the DNA-HuAd5 vaccine and was statistically significant (p = 0.0407) compared to the negative controls. In study 3, the efficacy of the DNA-HuAd5 vaccine was also low and was not statistically significant (p = 0.2222) compared to the negative controls. The efficacy of the DNA-GC46 vaccine, however, was very high (93% protection) and was highly statistically significant (p < 0.0001) compared to the negative controls.

**Fig. 4:**
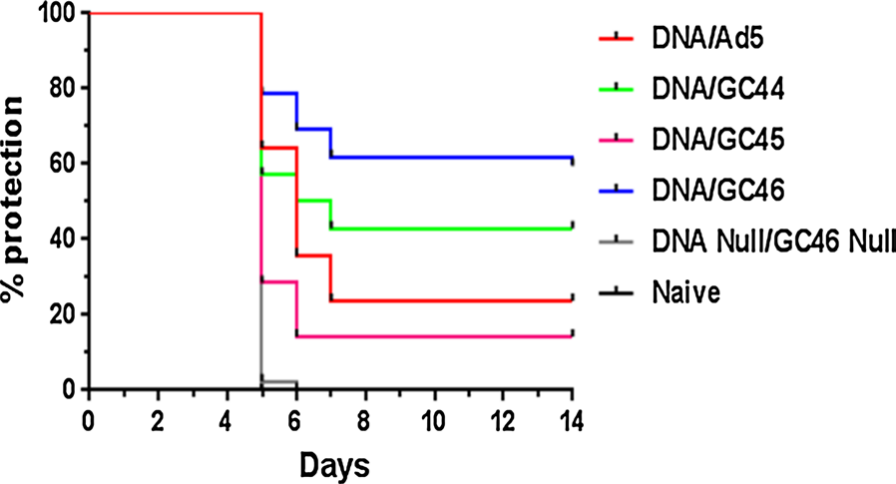
Time to patency in protection studies 1–3. Patency data from all three protection studies is presented in a Kaplan–Meier graph. Parasitaemia was evaluated on days 5, 6, 7, 9 and 14 post-challenge. Thirty fields, representing approximately 30,000 red blood cells (RBC), were examined for each slide. A mouse was considered positive if a single parasitized RBC was observed

In each study, the DNA-GC46 vaccine protected a higher percentage of mice than the DNA-HuAd5 vaccine, however the difference was most striking in study 3. When the data from all three studies were combined, the DNA-HuAd5 vaccine protected 10 of 42 mice (24% protection), while the DNA-GC46 vaccine protected 25 of 42 mice (60% protection). The protection induced by the DNA-HuAd5 vaccine (p = 0.0011) and the DNA-GC46 vaccine (p < 0.0001) were both statistically significant compared to the negative controls. Moreover, when compared to each other, the efficacy of the DNA-GC46 vaccine was significantly higher than the DNA-HuAd5 vaccine (p = 0.0018). All of the vaccine groups also showed a modest delay to patency relative to the negative controls (Fig. 4). The time to patency, however, was not significantly different between the vaccine groups. These findings demonstrate that the GC46 vector conferred a higher level of protection than the HuAd5 vector.

### DNA-GC vaccines induce robust T cell and antibody responses in mice

**Fig. 5:**
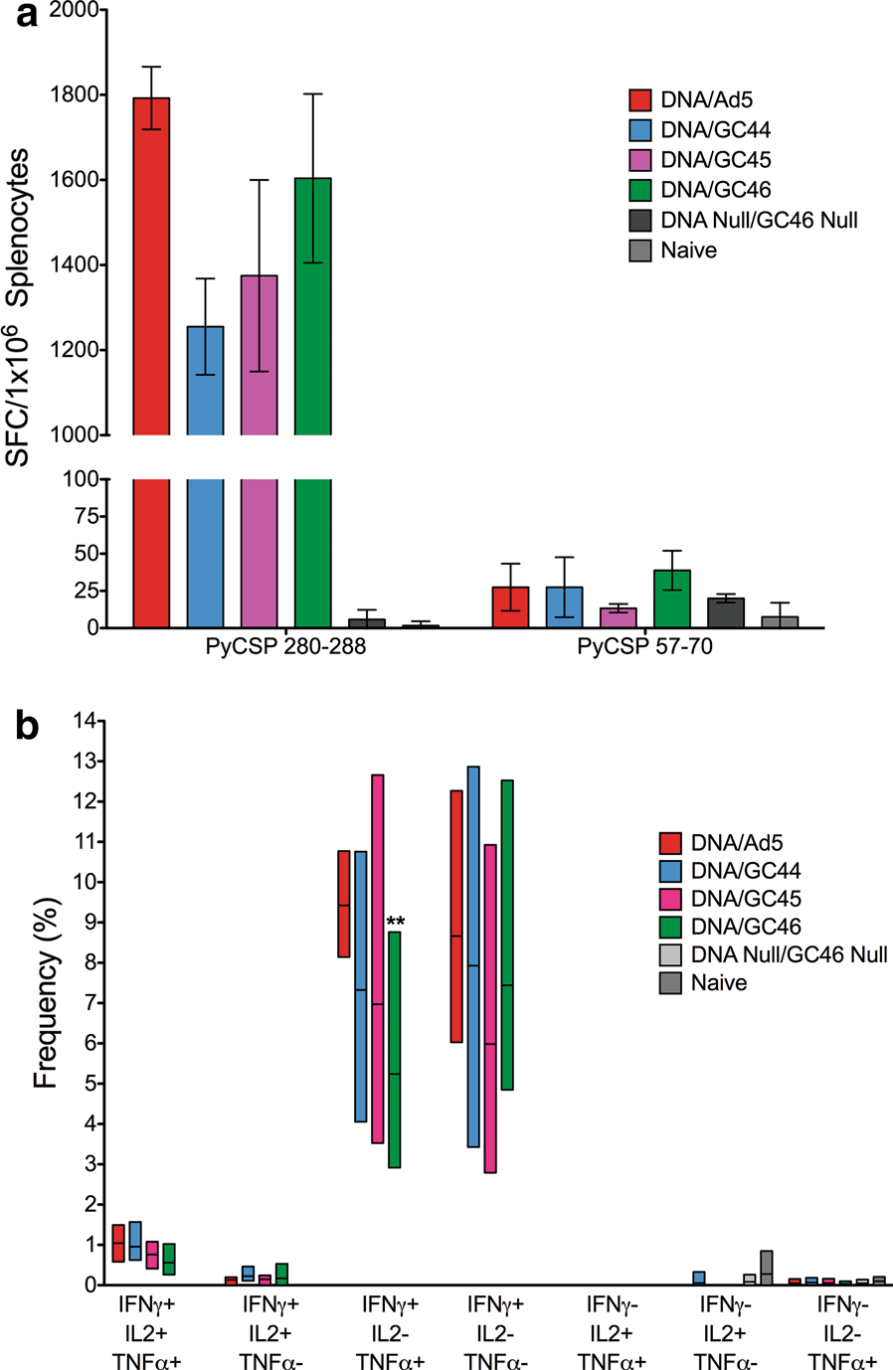
T cell responses induced by the prime-boost vaccines in protection study 1. IFN-γ ELISpot and ICS responses were assessed 12 days after the adenovirus boost. **a** IFN-γ ELISpot. Splenocytes from group pools (200,000 cells/well) were stimulated with A20.2J cells loaded with peptides encoding the *Py*CSP immunodominant CD8^+^ T cell epitope (amino acids 280–288) or the *Py*CSP CD4^+^ immunodominant/nested CD8^+^ subdominant T cell epitopes (amino acids 57–70). Spot forming cells (SFC)/1 × 10^6^ splenocytes are presented. *Bars* represent the mean ± standard deviation from quadruplicate wells. **b** ICS. Splenocytes from individual mice (6 mice/group) were stimulated with A20.2J cells loaded with a peptide encoding the *Py*CSP immunodominant CD8^+^ T cell epitope. Boolean gating was used to define the frequency of antigen-experienced CD8^+^ T cells (CD19^−^/NKp46^−^/CD3^+^/CD8^+^/CD44^+^ “bright”) expressing IFN-γ, IL-2 and/or TNF-α. The *line within the box* represents the mean and the *bottom and top lines of the box* represent the minimum and maximum responses observed in individual mice. **indicates statistical significance compared with the DNA-HuAd5 vaccine

The immunogenicity outcomes of protection study 1 are presented in Fig. 5. The *Py*CSP-specific cellular immune responses were assessed from splenocytes harvested 12 days after the adenovirus boost using IFN-γ ELISpot and ICS by flow cytometry. The IFN-γ ELISpot response to stimulation with the *Py*CSP CD4^+^ immunodominant/nested CD8^+^ subdominant epitopes (amino acids 57–70) were very low (mean < 50 sfc/m) in all four vaccine groups (Fig. 5a). In contrast, the responses to stimulation with the *Py*CSP immunodominant CD8^+^ T cell epitope (amino acids 280–288) were much higher (mean >1200 sfc/m) in all four vaccine groups. The IFN-γ ELISpot responses in the DNA-HuAd5 and DNA-GC46 groups were particularly robust, with mean spot forming cells and standard deviations of 1793 ± 74 and 1604 ± 198 sfc/m, respectively.

Further phenotypic and functional analysis of individual animals by ICS confirmed that the primary T cell populations responding to *Py*CSP 280–288 peptide stimulation were antigen-experienced CD8^+^ T cells (CD3^+^/CD8^+^/CD44^+^ “bright”) expressing IFN-γ (IFN-γ^+^/IL-2^−^/TNF-α^−^) or IFN-γ and TNF-α (IFN-γ^+^/IL-2^−^/TNF-α^+^) (Fig. 5b). Much lower frequencies of triple positive (IFN-γ^+^/IL-2^+^/TNF-α^+^) and IFN-γ/IL-2 double positive (IFN-γ^+^/IL-2^+^/TNF-α^−^) CD8^+^ T cells were observed. Overall, the frequency of the observed subsets was comparable in all vaccine groups, except that the DNA-GC46 vaccine induced a robust, but significantly lower frequency of IFN-γ^+^/IL-2^−^/TNF-α^+^ CD8^+^ T cells than the DNA-HuAd5 vaccine (DNA-GC46: 5.24% ± 2.09%; DNA-HuAd5: 9.42 ± 0.92%; p = 0.0043). Consistent with the IFN-γ ELISpot data, multifunctional T cell analysis indicated that cytokine expression from CD4^+^ T cells was negligible after stimulation with the *Py*CSP CD4^+^ immunodominant epitope (amino acids 57–70) (Additional file 1).

**Fig. 6:**
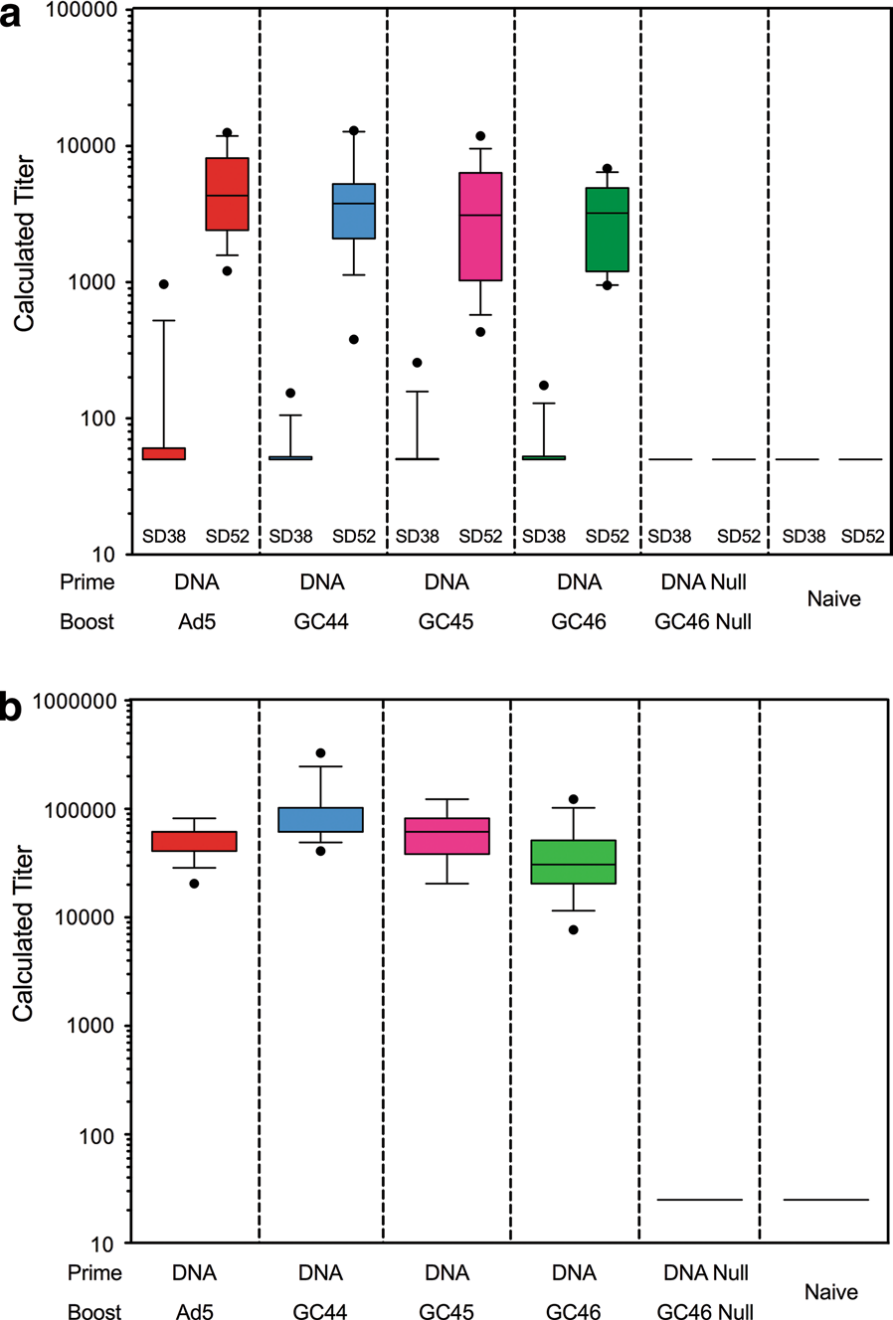
Antibody responses induced by the prime-boost vaccines in protection study 1. **a** ELISA. *Py*CSP-specific antibody responses (14 mice/group) were assessed on study day 38 (SD38) and study day 52 (SD52), 5 days before and nine days after the adenovirus boost. Plates were coated with a peptide encoding a portion of the immunodominant *Py*CSP repeat region (QGPGAP)_4_. The data are presented as a box-and-whisker plot. The *line within the box* represents the median, the *bottom* and *top lines of the box* represent the 25th and 75th percentiles, the *whiskers* represent the 10th and 90th percentiles and the *dots* represent outliers. The lowest dilution assayed was 1/50. **b** IFA. *Py*CSP-specific antibody responses (14 mice/group) were assessed on day 52, 9 days after the adenovirus boost. The data are presented as a box-and-whisker plot. The median and upper box lines overlap in the DNA-HuAd5 data and the median and lower box lines overlap in the DNA-GC44 data. The lowest dilution assayed was 1/40

Total IgG antibody responses to the *Py*CSP repeat region (QGPGAP)_4_ were assessed by ELISA with sera collected five days before (study day 38) and nine days after (study day 52) the adenovirus boost. In all four vaccine groups, responses were low after the DNA prime, but increased approximately 100-fold following the adenovirus boost (Fig. 6a). Following the adenovirus boost, the mean antibody titre of the DNA-HuAd5 group was higher than the titres of the DNA-GC groups although the differences were not statistically significant. IFA responses against *P. yoelii* sporozoites were evaluated with post-adenovirus boost sera. All four vaccine groups induced antibodies that recognized native *P. yoelii* parasites (Fig. 6b). In general, IFA titres were comparable in all vaccine groups.

## Discussion

The WHO has estimated that approximately 429,000 people died from malaria in 2015 [52]. Despite decades of effort, a highly effective malaria vaccine is not available. The leading vaccine candidate, RTS,S, has modest efficacy against clinical malaria in children and infants living in Africa [53, 54]. However, after 4 years, efficacy slowly wanes to zero [55, 56]. Based upon these results, new approaches are needed to meet the preferred product characteristics target of 75% efficacy [33].

Vector-based prime-boost vaccines are a compelling approach for malaria vaccine development. In addition to the positive protection data obtained with the DNA-HuAd5 vaccine candidate [5], a prime-boost regimen with chimpanzee adenovirus type 63 (ChAd63) and modified vaccinia Ankara (MVA) vectors expressing *Pf*CSP or a *P. falciparum* thrombospondin-related adhesion protein construct with a multi-epitope (ME) string of 17 *P. falciparum* B cell and T cell epitopes (*Pf*ME-TRAP) protected a total of 1 of 15 or 5 of 29 volunteers, respectively, against CHMI [6, 57]. In a Phase 2b trial, the ChAd63-MVA *Pf*ME-TRAP vaccine reduced the risk of malaria infection in Kenyan adults by 67% during the first 8 weeks post-immunization [58]. Although this vaccine does not meet the 75% efficacy target, these results are very encouraging and suggest that a non-human primate adenovirus vector can be a component of an efficacious malaria vaccine.

The major rationale for this study was to develop highly potent adenovectors with low human seroprevalence as an alternative to HuAd5, as high seroprevalence in malaria-endemic countries may compromise the efficacy of a HuAd5-based vaccine. The data presented in this report support the development of three new adenovirus vaccine vectors, GC44, GC45 and GC46. These vectors were isolated from wild gorillas in Rwanda and cluster by phylogeny in the same subgroup (species C adenoviruses) as two other potent vaccine vectors, HuAd5 and ChAd3. The results presented indicate that the seroprevalence of NAb specific for GC44, GC45 and GC46 are low in Kenya and Ghana, two malaria-endemic countries in sub-Saharan Africa. In fact, only 1–3% of adults living in Kenya and Ghana have NAb titres against GC44, GC45 or GC46 that are >200, a titre that has been shown to affect the potency of HuAd5-based vectors in previous clinical trials [41]. Moreover, since the seroprevalence and NAb titres against adenoviruses are lower in children than adults [37], pre-existing immunity should not affect the potency of GC-based vaccines in pediatric populations.

The superior performance of GC46 over HuAd5 highlights its potential for malaria vaccine development. Efficacy studies in the *P. yoelii*/mouse model indicated that a prime-boost regimen with DNA-*Py*CSP and GC-*Py*CSP vectors could protect mice against a *P. yoelii* challenge. GC46 was more protective than HuAd5 in three separate experiments and when analysed together, the efficacy of GC46 was significantly higher than HuAd5 (p > 0.0018). Typical of complex biological systems, overall efficacy levels varied between experiments. Results also indicated that the GC-*Py*CSP vectors are more immunogenic than HuAd5-*Py*CSP following a single immunization. At the lower doses, the three GC-*Py*CSP vectors all induced higher CD8^+^IFN-γ^+^ T cell responses than HuAd5-*Py*CSP, and at the highest dose, the three GC-*Py*CSP vectors all induced higher antibody responses than HuAd5-*Py*CSP. GC46-*Py*CSP, in particular, was highly immunogenic, inducing greater T cell and antibody responses than HuAd5-*Py*CSP at all doses. Similar results were obtained with GC44, GC45 and GC46 vectors expressing respiratory syncytial virus (RSV) [38], herpes simplex virus 2, Epstein Barr virus, and other malaria antigens (unpublished data). These results highlight the versatility of these new gorilla adenovirus vectors for vaccine development.

Previous studies with DNA and adenovirus-based vaccines in the *P. yoelii*/mouse model have indicated that protection is dependent on CD8^+^ T cells [16, 22]. CD8^+^ T cell responses have also been associated with the protection elicited by the DNA-HuAd5 and ChAd63-MVA malaria vaccines in humans [5, 6]. The GC-*Py*CSP vectors evaluated in this report all induced robust CD8^+^ T cell responses after priming with DNA. Phenotypic and multifunctional analysis identified antigen experienced CD8^+^IFN-γ^+^ T cells or CD8^+^IFN-γ^+^TNF-α^+^ T cells as the primary cellular subsets responsible for this response. Interestingly, the only significant immunological difference observed between the DNA-HuAd5 and DNA-GC46 regimens was in the frequency of CD8^+^IFN-γ^+^TNF-α^+^ T cells. These analyses suggest that immune parameters other than IFN-expressing CD8^+^ T cells may be involved in protective immunity. Overall, the DNA-GC46 vaccine induced robust CD8^+^ IFN-γ^+^ T cell and antibody responses, as well as consistently significant protection, thereby indicating that GC46 is a compelling alternative to HuAd5 for clinical development.

The capacity of the GC vectors to induce robust and protective immune responses suggests that GC vectors belong to a select group of adenovirus vectors that are at least on par with HuAd5 in terms of potency. These results are encouraging because most adenovirus vectors, with the exception of ChAd3, are less immunogenic than HuAd5 [39, 43–46]. For example, preclinical data demonstrate that a single administration of 1 × 10^10^ viral particles of a ChAd3-Ebola virus vector protected macaques from Ebola virus challenge [46]. This is similar to what has been observed with a HuAd5-Ebola virus vector [59]. Importantly, none of the other leading adenovectors (human adenovirus serotype 35, human adenovirus serotype 26 or ChAd63) were capable of providing this high-level protection [44, 46]. As a result, ChAd3 is currently being tested in clinical trials as a vaccine vector for Ebola virus and hepatitis C virus (HCV) [60–63].

## Conclusions

The low seroprevalence, robust immunogenicity and enhanced efficacy of GC46 vectors relative to HuAd5 vectors suggest that this gorilla-based adenovector has key performance characteristics for an effective malaria vaccine. A DNA-GC46 malaria vaccine may be more efficacious than a DNA-HuAd5 malaria vaccine in humans, especially those individuals with high HuAd5 NAb titres. Additional efficacy, if needed, could potentially be obtained by incorporating new protective antigens into the vaccine [6, 64] or by improving the immunogenicity of the individual DNA or adenovirus vaccine components [65, 66].

## Additional file

**Additional file 1.** CD4^+^ T cell responses induced by the prime-boost vaccines in protection study 1.
