# Supplementary figures and images for: New gorilla adenovirus vaccine vectors induce potent immune responses and protection in a mouse malaria model
Source: Malar J. 2017 Jul 3;16:263. doi: 10.1186/s12936-017-1911-z (PMC5496260; doi:10.1186/s12936-017-1911-z)

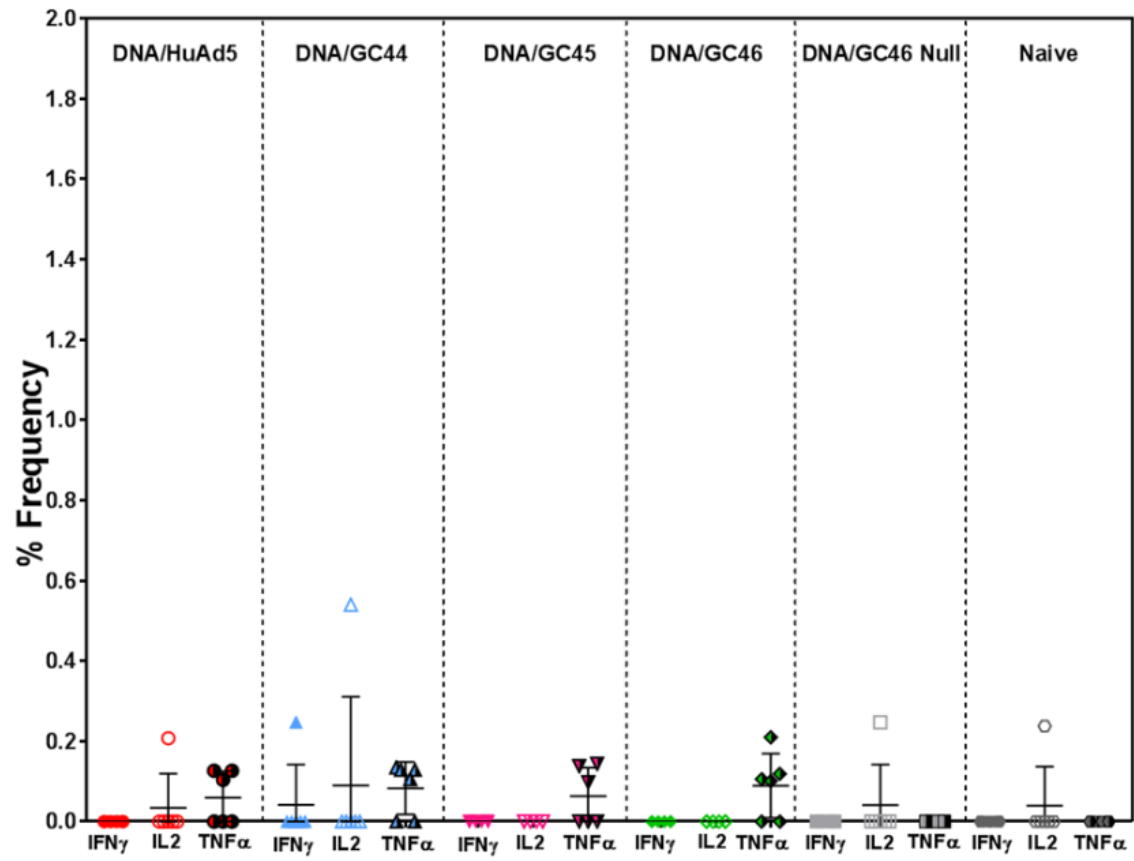

Supplement: Supplementary file 1 — Additional file 1. CD4+ T cell responses induced by the prime-boost vaccines in protection study 1. [file 12936_2017_1911_MOESM1_ESM.pdf]
